# Objective Assessment of Hypernasality in Patients with Cleft Lip and Palate with the NasalView System: A Clinical Validation Study

**DOI:** 10.1155/2012/321319

**Published:** 2012-02-12

**Authors:** Kai Wermker, Susanne Jung, Ulrich Joos, Johannes Kleinheinz

**Affiliations:** ^1^Department of Cranio-Maxillofacial Surgery, Fachklinik Hornheide, 48157 Muenster, Germany; ^2^Department of Cranio-Maxillofacial Surgery, Research Unit Vascular Biology of Oral Structures (VABOS), University Hospital Muenster, 48149 Muenster, Germany

## Abstract

*Introduction*. The objective of this investigation was to evaluate the reliability and validity of the NasalView system as a screening tool for hypernasality within the scope of a routine diagnostic procedure in cleft lip and palate patients. *Material and Methods*. In a collective of 95 patients with cleft and lip palate ranging from 4 to 25 years of age, hypernasality was exploited perceptually, patients were classified in four degrees, and nasalance was measured objectively with the NasalView system. Speech stimuli existed in one nasal and one nonnasal sentence; nasalance ratio and distance were calculated. *Results*. The test-retest error was within a range of 2%. Sensitivity ranged from 83.3% to 91.1% for the nonnasal sentence, from 70% to 78.4% for nasalance ratio and from 68.1% to 81.1% for nasalance distance. Specifity ranged from 87% to 93.1% for the nonnasal sentence, from 69.6% to 97.5% for nasalance ratio, and from 70.7% to 73.9% for nasalance distance. *Conclusions*. With a quick and gentle screening procedure, it is easily possible to identify hypernasal patients by an objective diagnostic tool of hypernasality, the NasalView system, with good reliability and validity.

## 1. Introduction

The most common speech impairment of patients with cleft lip and palate are resonance disorders [1]. They are characterized as impairments of the balance between oral and nasal acoustic energy and occur in form of hypernasality, hyponasality and mixed forms, for example, the cul-de-sac resonance (Vrticka [2–5], Bressmann and Sader [6]). Hypernasality due to velopharyngeal insufficiency in patients with cleft lip and palate can be caused by structural inadequacy and functional incompetence [6]. In some cases even after anatomically surgical reconstruction of the soft and the hard palate and consequent logopaedic treatment, hypernasality remains. Velopharyngeal morphology and function and perceptual consequence have to be assessed objectively to decide on a surgical intervention, for example, velopharyngoplasty to improve speech capability.

Regular speech and resonance disorders are evaluated by perceptual assessment; the quality of judgement depends on the listeners experience and academic training (Lewis et al. [7]). Because of this interrater variability, it is useful to augme that the subjective assessment by an objective quantitative instrumental analysis of resonance disorders.

An instrumental measurement of hypernasality can be performed by the Kay Nasometer (Kay Elemetrics, Lincoln Park, NJ, USA) and with the NasalView system (Tiger Electronics, Seattle, WA, USA), developed by Awan in 1997 [8]. Both systems measure the nasalance by calculating the proportion of the nasal energy in speech from separate measurements of nasal and oral sound pressure level, nasalance = nasal/[nasal + oral] × 100% (Fletcher [9, 10]).

For the Nasometer already several validation studies exist, demonstrating the good correlation between perceptual judgement of oronasal sound balance and nasalance measurement. In addition, they present solid validation data such as sensitivity, specificity, and efficiency (Dalston et al. [11, 12], Hardin et al. [13], Nellis et al. [14], Watterson et al. [15, 16], and Stellzig et al. [17]).

Bressmann et al. [18–21] established a solid validation for the NasalView System in German speaking cleft patients. Depending on the measurement, nonnasal sentences, nasalance distance and nasalance ratio, and depending on the differentiation of patients with different levels of hypernasality, in their investigations the sensitivity ranged from 70.7% to 91.1%, the specificity ranged from 73% to 97.1%, and the efficiency ranged from 71.4% to 91.5%.

The objective of this prospective clinical validation study was to evaluate practicability, reliability, and validity of the NasalView system as a noninvasive screening tool for resonance disorders in the course of a routine diagnostic for patients with cleft lip and palate.

## 2. Material and Methods

### 2.1. Subjects

100 patients with cleft lip and palate were examined. Three patients were excluded from the analysis due to reduced cooperation and compliance, in two cases the data had to be rejected due to inaccurate recording.

A total of 95 patients from 4 to 25 years of age (median age 9.25 years ± 4.25) were analysed in this investigation. Of these 52 females and 43 males, 54 had a complete unilateral cleft of the lip, alveolus and palate, 12 had an bilateral cleft lip, alveolus, and palate, 19 a cleft of the palate, and 10 patients suffered from velopharyngeal insufficiency in combination with minor forms of clefting (submucous cleft and uvula bifida).

### 2.2. Objective Measurement of Nasalance

Nasalance was measured with the NasalView system, version 1.2, Tiger Electronics DRS Inc., Seattle, WA, USA. The measuring was performed following standard procedures in a noiseless room. Before each recording the NasalView was calibrated according to the manufacturer instructions. The test items were recorded at a sampling rate of 22.5 Hz stereo, 16 bit; a generic IBM compatible PC with an Intel Pentium IV Processor and a Soundblaster Audio PCI 128 soundcard (Creative Labs, Singapore) were used.

For each test item the NasalView measures the following parameters of nasalance.

**Figure 1:**
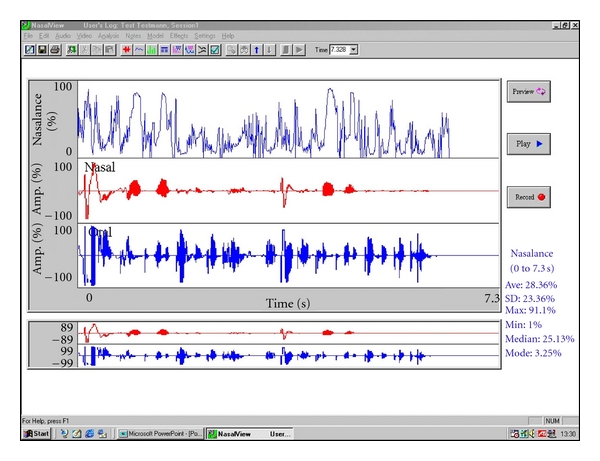
Sample screenshot of the NasalView system showing oszillograms for oral, nasal, and nasalance curves recorded realtime and nasalance statistic for a nonnasal test item.

The average nasalance (Ave, i.e., the arithmetic mean), the standard deviation (SD), maximum (max) and minimum (min), the median of nasalance (Median), and mode.  Figure 1 illustrates a sample.

In our study the nasalance values Ave and Median were analysed. The other parameters (mode, maximum, minimum, and standard deviation) were analyzed in other studies before and were not reliable enough to allow for conclusive results for there is no sharp distinction between nasal and nonnasal test items (Bressmann et al. [18–21]). Even in the same patient they show similar results in nonnasal and nasal speech stimuli, so a clear distinction between hypernasal and normal speech is clearly impossible.

The nonnasal sentence (nnS-testitem) “Der Peter trinkt die Tasse Kakao” (“The Peter drinks a cup of cocoa”) and the nasal sentence (nS-testitem) “Mama und Nina naschen Marmelade” (“Mama and Nina nibble marmalade”) served as speech stimuli. Other investigators had underlined the importance of adjusting the level of complexity to the developmental stage of the examined children (Van Denmark and Swickard [22], Baker et al. [23]). The chosen two sentences are simple enough to be recorded easily from children from the age of four years on. In addition, the results of other studies underlined the efficiency of a stimulus length of six syllables or a shortened recording procedure [21, 24].

### 2.3. Perceptual Analysis

Perceptual assessment of nasality was achieved by samples of spontaneous speech during a semistandardized interview routine by an experienced speech therapist. The degree of hypernasality was then classified as normal (no hypernasality), borderline (mild hypernasality), marked (hypernasality), and severe (impaired understanding). Patients were then grouped according to results of the perceptual assessment.

### 2.4. Statistical Analysis

Test items were recorded twice with the NasalView system during one examination; the arithmetic means and standard deviations of the difference between the nasalance measuring were calculated to determine the test-retest error for both, the Ave-nasalance value and the Median-nasalance value. To decide which nasalance value was more reliable, Ave or Median, the differentiation between both values was tested on significance by using the Wilcoxon matched pairs signed rank test.

When a sentence was recorded more than once, the arithmetic mean was calculated and valued for this test item. For each patient nasalance distance (maximum nasalance–minimum nasalance) were calculated (Bressmann et al. [21]).

For each group of patients classified according to the perceptual assessment of four degrees of hypernasality, arithmetic means and standard deviation for the various measures were calculated. Differences among the groups were tested on significance using ANOVA and the post hoc Scheffé tests.

In diagnostics high sensitivity, specificity, and efficiency are required. Sensitivity in our case is the percentage of patients correctly identified by the NasalView system as hypernasal in relation to all hypernasal patients. Specificity is defined as percentage of patients with no perceptual hypernasality in relation to all patients with regular nasalance parameters measured by the NasalView system. Efficiency or overall diagnostic accuracy is defined as the number of all correct decisions over the number of all decisions. An apt tool to achieve optimum values are Receiver-Operating Characteristics (ROC) curves that plot the sensitivity and false positive rate [25, 26]. With these ROC curves, validation data was determined for the various discriminations between degrees of hypernasality.

## 3. Results

### 3.1. Reliability

A low test error stands for good reliability and is a prerequisite for the routine application of a diagnostic tool. A total sum of 157 pairs of sentences were analysed to determine the test-retest error.  Table 1 presents the results.

Because the test-retest error for Ave-nasalance values are statistically significantly lower than the test-retest error for Median-nasalance values (*P* < 0.001), only Ave values are reported as nasalance value in the following results.

### 3.2. Group Differences

According to the results of the perceptual analysis, 23 patients were classified normal, 35 patients were borderline cases, 27 patients showed marked hypernasality, and 10 patients had severe hypernasal speech.

Table 2 provides the results of the objective computerized measurements with the NasalView system. With the exception of the nasalance of the nnS-test item (nonnasal sentence) the differences between the single groups were significant at the *P* < 0.05 level. For the nonnasal sentence group, differences were significant with *P* < 0.01.

### 3.3. Validation Data

The optimum cutoff, sensitivity, specificity, and efficiency were calculated for the various measurements regarding the following discriminations:

- differentiation between all patients with regular resonance and all hypernasal patients (Table 3);
- differentiation between all patients with regular resonance and hypernasal patients with marked and severe hypernasality excluding borderline cases (Table 4);
- differentiation between patients with marked and severe hypernasality (Table 5);
- differentiation between patients with no or borderline rhinophonia and hypernasal patients with marked and severe hypernasality (Table 6). This discrimination is based upon the therapeutic consequence: in cases of marked and severe hypernasality, treatment of the speech disorder—speech therapy or surgical intervention is necessary, whereas in borderline cases the further clinical development is scrutinized.

## 4. Discussion

The application of the NasalView system was easy due to the fact that the measuring of nasalance is entirely noninvasive. In addition to the practicability, this investigation also demonstrates the good reliability of computerized assessment of hypernasality with the NasalView system. In accordance with Awan [8], who accounted for a test-retest error of 2% for the NasalView system and a test-retest error of 3% for the Nasometer in a study with 20 adult subjects, we established a test-retest error under 2% when the average value of the reported nasalance statistic was used. This small error implicates a good reliability of the measuring technique.

The validation data established in our investigation are comparable to the results of other studies dealing with the NasalView system or the Nasometer.

For the Nasometer, Dalston et al. [11, 12] and Hardin et al. [13] reported an efficiency ranging from 79.2% to 93% depending on whether borderline cases were included or not. In 30 German speaking cleft patients with a mean age of 13.5 years, Stellzig et al. measured in 1994 an overall diagnostic accuracy of 90% for the Nasometer [17]. Nasalance measurement with the NasalView system and the Nasometer differ technically and in appliance. Therefore, a direct comparison of the detailed results is not possible (Lewis and Watterson [27]).

As far as the NasalView system is concerned comparable investigations have been performed by Bressmann, who analysed nasalance measurements of 133 and 140 German speaking patients with cleft lip and palate from 10 to 66 years of age, mean age = 17 years (Bressmann et al. [20, 21]).

When differentiating between healthy subjects and hypernasal patients, we found the same optimum cutoff of 28.5% for the nonnasal test item. Whereas Bressmann reported an efficiency of 73%, we found a higher overall diagnostic accuracy of 84.2% for this test item and the discrimination. For the nasalance ratio and nasalance distance in our study, we found lower values of 70.5% and 69.5% than Bressmann, who reported an accuracy of ranging from 75.2% to 77.4%. When excluding borderline cases from the analysis, sensitivity, specificity, and efficiency improve considerably. In the present study for the nonnasal test item efficiency improved to 91.7% compared to 81.4% found by Bressmann et al. [20]. Although efficiency for nasalance ratio and distance improved to 85% and 78.3%, the results are lower than the values measured by Bressmann et al. [21].

In our data, optimum cutoffs for the nasalance ratio were higher and cutoffs for the nasalance distance were lower than in the comparable validation study [21]. These discrepancies can be provoked by the influence of a different dialect, as described by Van Lierde et al. [28], or the use of similar but not identical sentences.

Another decision of clinical importance is the differentiation between patients with no and mild hypernasality, normal and borderline cases, and patients with marked and severe rhinophonia. Due to clinical experience, mostly a mild hypernasality does not impair intelligibility of speech and represents no stigma for the patient in his social environment. In most cases of borderline hypernasality, no treatment is mandatory. On the other hand, in patients who suffer from marked or severe hypernasality with impaired speech regularly treatment, that is, surgical intervention with velopharyngoplasty is necessary. Differentiating between patients with the need for intervention and those who do not need therapy as described above, again the best efficiency of 90.5% results from the nonnasal sentence with a cutoff of 33%. When using nasalance ratio and distance, we found a diagnostic accuracy of 82.1% and 72.6%, respectively, for the differentiation between patients with no and borderline hypernasality on the one side and patients with marked and severe hypernasality on the other side. This clear differentiation between hypernasality with the need for treatment and no or mild hypernasality without need of treatment need was not yet described in previous studies.

## 5. Conclusions

The results of this clinical validation identify the NasalView system as an apt and userfriendly device to identify patients with resonance disorders. A short screening procedure including a nasal and a nonnasal test item is sufficient to differentiate between the perceptual degrees of hypernasality with good reliability and validity.

To come to a sensible decision as far as the further treatment of patients with cleft lip and palate and resulting hypernasality is concerned, it is necessary to assess the velopharyngeal sphincter with regard to its structure, function, and the perceptual consequences. The validation data of our investigation among others indicates that an objective measuring of rhinophonia is helpful to supplement the perceptual assessment of hypernasality by an experienced speech therapist.

We consider the objective computerized diagnostic of nasality with the NasalView system an expedient completion and helpful screening tool for resonance disorders, but not as a replacement of perceptual judgements by experienced speech therapists and listeners.

## Figures and Tables

**Table 1:** Test-retest errors for ave and median nasalance measurement.

Measurement	Pairs of test items		Total (*n* = 157)
	Nasal sentences (*n* = 95)	Nonnasal sentences (*n* = 62)	
D-Ave [%]			
Mean	1.50	1.37	1.44
SD	0.99	1.15	1.05
D-Median [%]			
Mean	2.84	1.66	2.37
SD	2.31	1.35	2.06

D-ave: difference of ave nasalance values between two test items; D-median: difference of median nasalance values between two test items mean: arithmetic mean; SD: standard deviation.

**Table 2:** Results grouped in different degrees of hypernasality.

Measurement	degree of hypernasality				*P*
	Normal (*n* = 23)	Borderline (*n* = 35)	Marked (*n* = 27)	Severe (*n* = 10)	
Nasalance of nS [%]					
Mean	44.08	45.06	47.65	55.30	0.048*
SD	4.63	6.12	5.66	5.42	
Nasalance of nnS [%]					
Mean	26.30	29.80	35.36	45.21	0.007**
SD	2.93	3.49	5.21	4.25	
Nasalance ratio					
Mean	0.602	0.671	0.745	0.819	0.036*
SD	0.081	0.139	0.142	0.085	
Nasalance distance [%]					
Mean	17.76	15.26	12.29	10.08	0.033*
SD	4.85	5.94	5.04	3.43	

nS: nasal sentence; nnS: nonnasal sentence; mean: arithmetic mean; SD: standard deviation *P*: *P* value of ANOVA (differences between degrees of hypernasality)*: *P* < 0.05  **: *P* < 0.01.

**Table 3:** Validation data for differentiation between patients with regular resonance and hypernasal patients.

Measurement	Cutoff	Sensitivity [%]	Specificity [%]	Efficiency [%]
Nasalance of nnS	28.5%	83.3	87.0	84.2
Nasalance ratio	0.66	70.8	69.6	70.5
Nasalance distance	15.3%	68.1	73.9	69.5

nnS: nonnasal sentence.

**Table 4:** Validation data for differentiation between patients with regular resonance and hypernasal patients excluding borderline cases.

Measurement	Cutoff	Sensitivity [%]	Specificity [%]	Efficiency [%]
Nasalance of nnS	28.6%	91.9	91.3	91.7
Nasalance ratio	0.72	78.4	95.7	85.0
Nasalance distance	15.0%	81.1	73.9	78.3

nnS: nonnasal sentence.

**Table 5:** Validation data for differentiation between patients with marked and severe hypernasality.

Measurement	Cutoff	Sensitivity [%]	Specificity [%]	Efficiency [%]
Nasalance of nnS	40.0%	90.0	88.9	89.2
Nasalance ratio	0.818	70.0	77.8	75.7
Nasalance distance	10.5%	70.0	59.3	62.2

nnS: nonnasal sentence.

**Table 6:** Validation data for differentiation between patients with no and borderline rhinophonia and patients with marked and severe hypernasality.

Measurement	Cutoff	Sensitivity [%]	Specificity [%]	Efficiency [%]
Nasalance of nnS	33.0%	86.5	93.1	90.5
Nasalance ratio	0.72	78.4	84.5	82.1
Nasalance distance	14.02%	75.7	70.7	72.6

nnS: nonnasal sentence.
